# Association between Gestational Weight Gain and Risk of Hypertensive Disorders of Pregnancy among Women with Obesity: A Multicenter Retrospective Cohort Study in Japan

**DOI:** 10.3390/nu15112428

**Published:** 2023-05-23

**Authors:** Momoka Ito, Hyo Kyozuka, Tomoko Yamaguchi, Misa Sugeno, Tsuyoshi Murata, Tsuyoshi Hiraiwa, Fumihiro Ito, Daisuke Suzuki, Toma Fukuda, Shun Yasuda, Keiya Fujimori, Yasuhisa Nomura

**Affiliations:** 1 Department of Obstetrics and Gynecology, Ohta Nisinouchi Hospital, 2-5-20, Nishinouchi, Koriyama City 963-8558, Fukushima, Japan; 2 Department of Obstetrics and Gynecology, Fukushima Medical University, 1, Hikarigaoka, Fukushima City 961-8141, Fukushima, Japan; tuyoshim@fmu.ac.jp (T.M.); fujimori@fmu.ac.jp (K.F.); 3 Department of Obstetrics and Gynecology, Iwase General Hospital, 20, Kitamachi, Sukagawa City 962-8503, Fukushima, Japan

**Keywords:** body mass index, gestational weight gain, obesity, preconception care, hypertensive disorders of pregnancy

## Abstract

The relationship between weight gain during pregnancy and the onset of hypertensive disorders of pregnancy in women with pre-pregnancy obesity remains unclear. We examined the effects of weight gain during pregnancy on hypertensive disorders of pregnancy among women with pre-pregnancy body mass index (BMI) ≥ 25.0 kg/m^2^. This multicenter retrospective cohort study included nullipara women who delivered at two units in Japan between 1 January 2013, and 31 December 2020. Singleton primipara (*n* = 3040) were categorized into two pre-pregnancy BMI groups: 25.0–<30.0, and ≥30.0 kg/m^2^. Using multiple logistic regression analyses (reported as adjusted odds ratio and 95% confidence interval), gestational weight gain effects on overall hypertensive disorders of pregnancy, gestational hypertension, and pre-eclampsia were determined. Gestational weight gain increased hypertensive disorders of pregnancy (1.09, 1.03–1.16, *p* < 0.05) and pre-eclampsia risk (1.10, 1.01–1.20, *p* < 0.05) among the BMI 25.0–<30.0 kg/m^2^ group and hypertensive disorders of pregnancy risk among the ≥30.0 kg/m^2^ group (1.07, 1.00–1.05, *p* < 0.05). Using receiver operating characteristic curve analyses, among the BMI 25.0–<30.0 kg/m^2^ group, for hypertensive disorders of pregnancy (area under the curve [AUC], 0.63, *p* < 0.05) and pre-eclampsia (AUC, 0.62; *p* < 0.05), the weight gain cut-off was 10.5 and 10.6 kg, with sensitivity/specificity of 0.47/0.73 and 0.50/0.73, respectively. For the BMI ≥30.0 kg/m^2^ group (AUC, 0.63, *p* < 0.05), the cut-off was 3.5 kg (sensitivity/specificity, 0.75/0.49). The optimal gestational weight gain for reducing hypertensive disorders of pregnancy among women with a pre-pregnancy BMI > 25 kg/m^2^ may facilitate personalized pre-conception counseling among women with obesity.

## 1. Introduction

Hypertensive disorders of pregnancy impact approximately 2.5% of all pregnancies in Japan [1]. Hypertensive disorders of pregnancy are the leading direct contributor to approximately 30,000 maternal deaths per year and cause 14% of maternal deaths worldwide [2,3], making their prevention a vital public concern within the field of obstetrics. Hypertensive disorders of pregnancy are commonly classified into pre-eclampsia and gestational hypertension, both of which involve the onset of hypertension at or after 20 weeks of gestation [4]. Pre-eclampsia is diagnosed when hypertension coexists with proteinuria or has indications of systemic illness, such as raised liver transaminase levels or thrombocytopenia. This multi-organ disorder affects various organs, including the kidneys, liver, and brain, and poses a significant cause of maternal and perinatal morbidity and mortality [4]. Gestational hypertension, another type of hypertensive disorders of pregnancy, presents as new-onset hypertension ≥20 weeks of gestation but without any organ dysfunction [5]. Hypertensive disorders of pregnancy and related conditions can lead to severe maternal and fetal complications [6], making it essential to formulate preventive strategies prior to pregnancy.

Maternal pre-pregnancy body mass index (BMI) and gestational weight gain are significant, modifiable factors affecting pregnancy outcomes, thereby underscoring the importance of preconception care. In the United States, 40% of females aged 20 to 39 years are diagnosed with BMI ≥ 30 kg/m^2^ [7,8], and nearly half of all women have a BMI of ≥25 kg/m^2^ at the onset of their pregnancies [9]. Likewise, in Japan, the growing prevalence of BMI ≥ 25 kg/m^2^ among women of reproductive age is raising concerns, echoing the trend seen in Western nations [10]. Given that a high body mass index (BMI) and substantial weight gain during gestation are significantly associated with an increased risk of developing hypertensive disorders during pregnancy [6,11,12], the management of pre-pregnancy BMI and careful control of weight gain during the course of pregnancy have emerged as crucial public health concerns. Addressing these issues could potentially serve as effective strategies to mitigate the incidence of hypertensive disorders during pregnancy, thereby improving the overall health outcomes for both mother and child [11,12].

Despite the high prevalence of obesity among women in the Fukushima Prefecture, Japan—the location of our research facility, where we find that 12.7% of women commence their pregnancies with a BMI of ≥25 kg/m^2^ [1]—the relationship between weight gain during pregnancy and the occurrence of hypertensive disorders of pregnancy among these obese women, especially those who already have a pre-pregnancy BMI of ≥25 kg/m^2^, remains inadequately elucidated in Japan and requires further exploration. Thus, we recommend, based on data from a single tertiary referral center, a maximum weight gain of 3.85 kg during pregnancy for primiparous women with a pre-pregnancy BMI of over 30 kg/m^2^ as a potentially preventive measure against hypertensive disorders of pregnancy [11]. However, our prior study had certain limitations, such as not categorizing hypertensive disorders of pregnancy into gestational hypertension and pre-eclampsia and only including women with a BMI over 30 kg/m^2^ due to the relatively small sample size.

In this study, we investigated the relationship between gestational weight gain and the incidence of hypertensive disorders of pregnancy, gestational hypertension, and pre-eclampsia among women with a pre-pregnancy BMI of 25 to <30 kg/m^2^ and ≥30 kg/m^2^ at multiple tertiary referral centers in Fukushima, Japan. Considering that the occurrence of hypertensive disorders of pregnancy is influenced by parity [11,13], our analysis focused on overweight and obese primiparous women.

## 2. Materials and Methods

### 2.1. Patients and Setting

We conducted this multicenter retrospective cohort research at two tertiary Maternal-Fetal Medical Units located in Fukushima Prefecture, Japan [14]. Participants included pregnant women who delivered at either facility between 1 January 2013 and 31 December 2020 [14]. The study adhered to the principles outlined in the Declaration of Helsinki. The study received approval from the Institutional Review Board of Ohta Nishinouchi Hospital (approval no. 42, as of 26 February 2021), and due to the retrospective nature of the study, informed consent was not required.

Data on maternal and obstetric outcomes were obtained from the hospital’s medical records. The collected maternal background data included maternal age at delivery, pre-pregnancy BMI, conception method, pre-pregnancy smoking habits, weight gain during pregnancy, and pre-pregnancy medical conditions such as hypertension and maternal diabetes mellitus (DM). Obstetric outcomes included hypertensive disorders of pregnancy, gestational hypertension, and pre-eclampsia. Women with multiple pregnancies, insufficient data, delivery before 22 weeks, chronic hypertension, and multiparous women were excluded.

### 2.2. Definition of Gestational Weight Gain

At the initial obstetric visit in our facility, all pregnant women are asked to provide their pre-pregnancy height and body weight, with this information being collected by professional midwives. Using these data, BMI was computed in accordance with the World Health Organization (Geneva) standards (body weight [kg]/height^2^ [m^2^]). Participants were classified into three group according to the BMI at the time of pregnancy as follows: BMI < 25.0, 25.0 to <30.0, and ≥30 kg/m^2^. Gestational weight gain was determined by subtracting the pre-pregnancy weight (kg) from the weight immediately prior to delivery (kg) [11,14,15].

### 2.3. Outcomes

Hypertensive disorders of pregnancy in our institution are defined as the onset of hypertension after the 20th week of gestation [11]. Hypertensive disorder was further categorized in pre-eclampsia and gestational hypertension. The phenotypes of pre-eclampsia and gestational hypertension are differentiated by the presence or absence of proteinuria, small for gestational age, or liver dysfunction [12].

### 2.4. Confounding Factors

In this analysis, we considered the following variables potential confounding factors: maternal age at delivery, maternal smoking status prior to pregnancy, maternal DM before pregnancy, and conception method. Maternal age was categorized into advanced maternal age (maternal age ≥ 30 years), or age < 30 years [16]. Conception methods such as in vitro fertilization, intracytoplasmic sperm injection, or the transfer of cryopreserved, frozen, or blastocyst embryos were classified under the umbrella of assisted reproductive technology (ART) [17]. The covariates’ selection criteria for the analysis model are established and based on our previous report in Japan [5,11,12,16].

### 2.5. Statistical Analysis

Maternal characteristics and obstetric outcomes were summarized according to the BMI categories (25.0 to <30.0, and ≥30.0 kg/m^2^). Student’s t-test was used to compare continuous variables, while chi-squared tests were used to compare categorical variables, between groups. The chi-squared test was used to analyze the categorical variable in obstetric outcome among BMI categories. To calculate the adjusted odds ratios (aORs) and 95% confidence intervals (CIs), multiple logistic regression models were developed. We utilized these models to evaluate the risk of hypertensive disorders of pregnancy connected to weight gain during pregnancy (continuous variable), taking into account factors such as maternal age (reference: <30 years), maternal smoking status, DM, and the method of conception (either with or without ART). A receiver operating characteristic curve was constructed due to the continuous nature of pregnancy weight gain to identify the cut-off values of outcomes significantly influenced by gestational weight gain, as determined by multivariable logistic regression analysis. The area under the receiver operating characteristic curve (AUC) was used to assess the diagnostic performance. The AUC is a measure used in statistics that represents the area underneath the receiver operating characteristic curve. An AUC close to 1 indicates a high reliability of the test. In our study, we determined the cut-off values based on the point where Youden’s index (the sum of sensitivity and specificity) reached its maximum. This index aids in identifying the optimal balance between sensitivity and specificity, thereby providing the most effective cut-off point for our analysis. IBM SPSS Statistics for Windows, version 29 (IBM Corp., Armonk, NY, USA) was employed for all statistical computations. A *p*-value less than 0.05 was considered to indicate statistical significance.

## 3. Results

**Figure 1:**
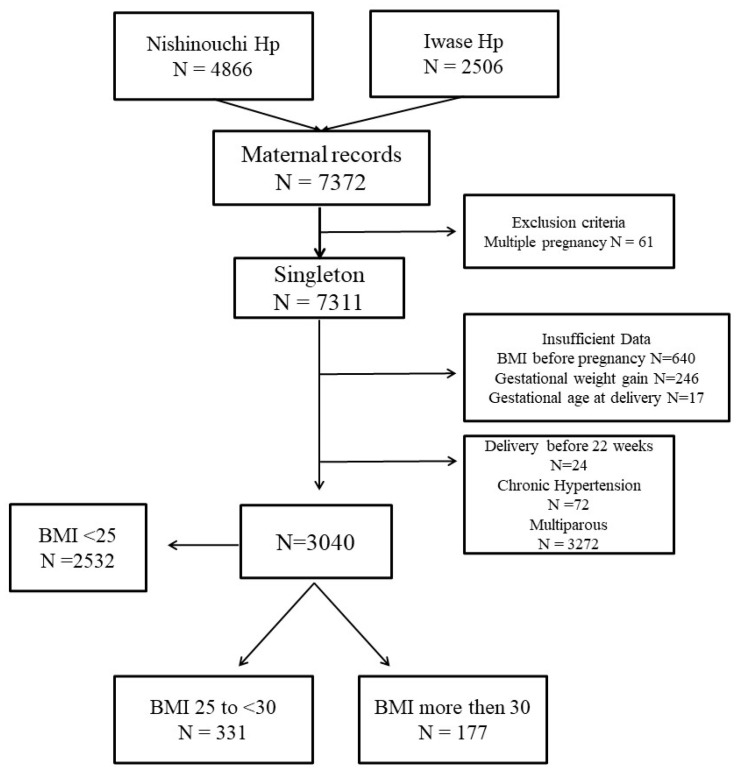
Patient flow for inclusion in the study and patient grouping. Women with multiple pregnancy, insufficient data, delivery before 22 weeks, chronic hypertension, multiparous and BMI < 25 Kg/m^2^ women were excluded. Hp, hospital; BMI, body mass index.

During the study period, there were 7372 neonatal records at Ohta Nishinouchi and Iwase Hospitals. Of these, 4332 cases were excluded based on our criteria. Consequently, 508 cases, including 331, and 177 with BMI 25 to <30, and ≥30 kg/m^2^ were eligible for inclusion in the present analysis (Figure 1).

Table 1 compares the maternal background and obstetric outcomes according to the BMI categories. Significant differences were observed in maternal background, regarding maternal DM before pregnancy (*p* < 0.001), and gestational weight gain (*p* < 0.001) between the two groups. No significant difference was observed in mean maternal age (*p* = 0.052), ART pregnancy (*p* = 0.199), maternal smoking status (*p* = 0.089), and maternal age of more than 30 years (*p* = 0.102) among the two groups. The chi-squared test showed that the occurrence of hypertensive disorders of pregnancy significantly increased along with the BMI categories (*p* < 0.017).

Table 2 summarizes the results of the multiple linear regression analysis of the association between gestational weight gain and hypertensive disorders of pregnancy (including gestational hypertension and pre-eclampsia) in women with a pre-pregnancy BMI of 25 to <30 and >30.0 kg/m^2^. Gestational weight gain was associated with the occurrence of hypertensive disorders of pregnancy (aOR: 1.09, 95% CI: 1.03–1.16, *p* < 0.01) and pre-eclampsia (aOR: 1.10, 95% CI: 1.01–1.20, *p* < 0.05) among women with a BMI of 25 to < 30 kg/m^2^. For the women with a BMI > 30 kg/m^2^, gestational weight gain was associated with the occurrence of hypertensive disorders of pregnancy (aOR: 1.07, 95% CI: 1.00–1.15, *p* < 0.05). The aOR (95% CI) for the association between gestational weight gain and pre-eclampsia risk among women with a BMI > 30 was 1.08 (0.99–1.18).

**Figure 2:**
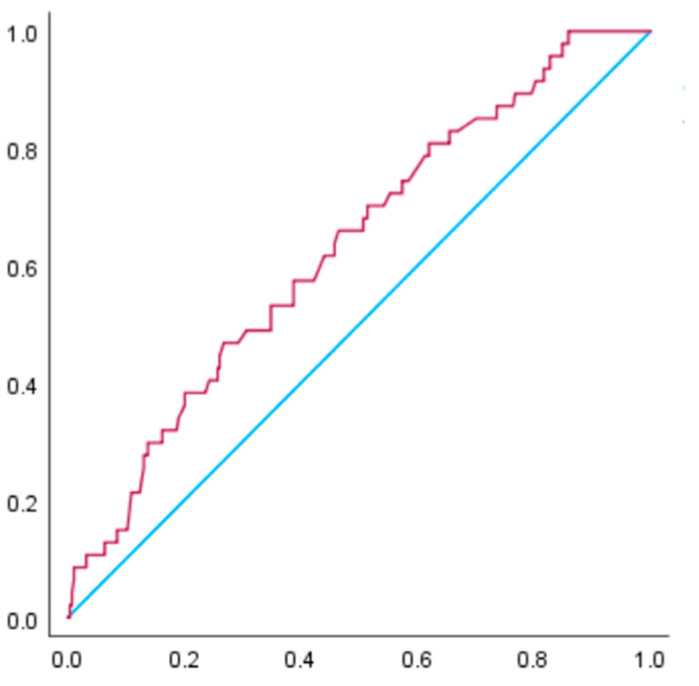
The receiver operating characteristic curve was constructed to examine the relationship between gestational weight gain and hypertensive disorders of pregnancy in women with a BMI of 25.0 to <30.0 kg/m^2^ before pregnancy. The area under the curve (AUC) is 0.63 (*p* < 0.05). The receiver operating characteristic curve analysis suggested that the cut-off value of weight gain during pregnancy for the occurrence of hypertensive disorders of pregnancy was 10.5 kg, with a sensitivity of 0.47 and specificity of 0.73.

**Figure 3:**
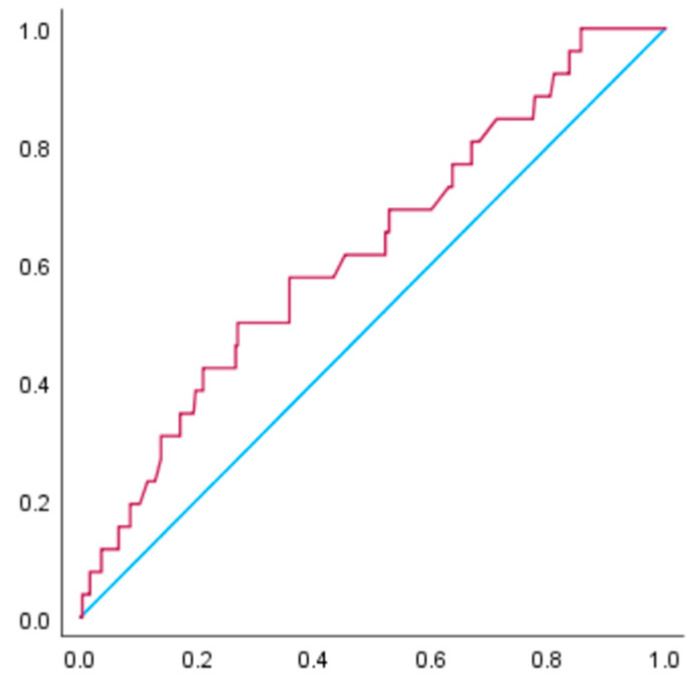
The receiver operating characteristic curve was constructed to examine the relationship between gestational weight gain and pre-eclampsia in women with a BMI of 25.0 to <30.0 kg/m^2^ before pregnancy. The AUC is 0.62 (*p* < 0.05). The receiver operating characteristic curve analysis revealed that the cut-off value of weight gain during pregnancy for the occurrence of pre-eclampsia was 10.6 kg, with a sensitivity of 0.50 and specificity of 0.73.

Figure 2 and Figure 3 illustrate the receiver operating characteristic curve for the prediction of hypertensive disorders of pregnancy among women with a BMI of 25 to <30 kg/m^2^. For these women, the AUC for gestational weight gain in relation to hypertensive disorders of pregnancy was 0.63 (95% CI: 0.55–0.71, *p* < 0.05), while the AUC for gestational weight gain in relation to pre-eclampsia was 0.62 (95% CI: 0.51–0.74, *p* < 0.05). The receiver operating characteristic curve analysis revealed that the cut-off value of weight gain during pregnancy for the occurrence of hypertensive disorders of pregnancy was 10.5 kg, with a sensitivity of 0.47 and specificity of 0.73. For the occurrence of pre-eclampsia, the cut-off value was 10.6 kg, with a sensitivity of 0.50 and specificity of 0.73.

**Figure 4:**
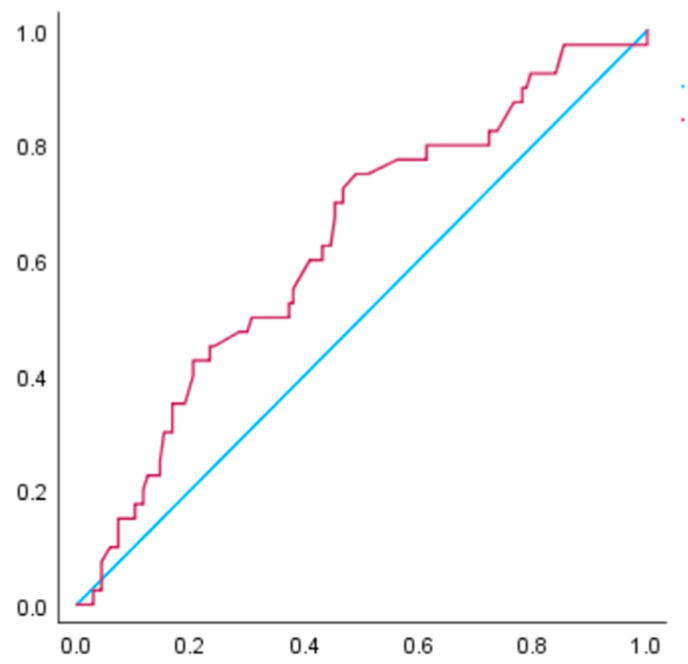
The receiver operating characteristic curve was constructed to examine the relationship between gestational weight gain and hypertensive disorders of pregnancy in women with a BMI of ≥30 kg/m^2^ before pregnancy. The AUC is 0.63 (*p* < 0.05). According to the receiver operating characteristic curve analysis, the cut-off value of weight gain during pregnancy for hypertensive disorders of pregnancy development was 3.5 kg, with a sensitivity of 0.75 and specificity of 0.49.

**Figure 5:**
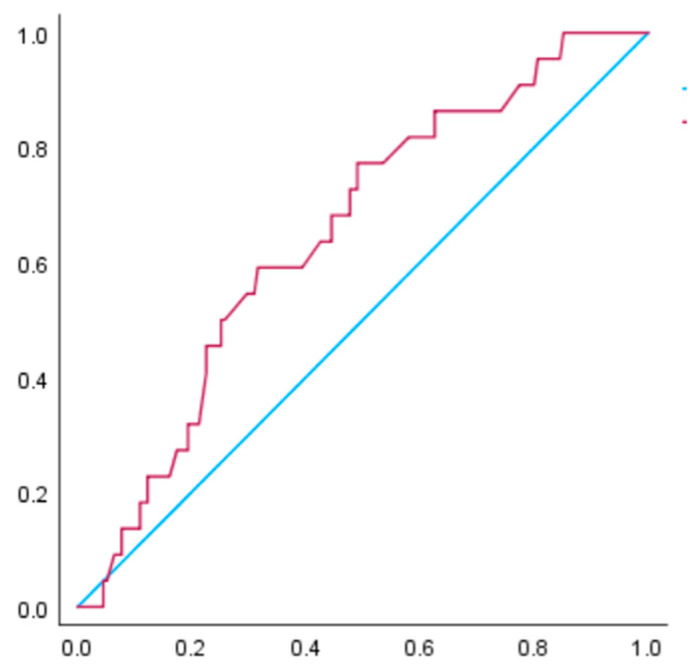
The receiver operating characteristic curve was constructed to examine the relationship between gestational weight gain and pre-eclampsia in women with a BMI ≥ 30 kg/m^2^ before pregnancy. The AUC is 0.65 (*p* < 0.05). According to the receiver operating characteristic curve analysis, the cut-off value of weight gain during pregnancy for pre-eclampsia was 3.6 kg, with a sensitivity of 0.77 and specificity of 0.49.

Figure 4 and Figure 5 illustrate the receiver operating characteristic curve for the prediction of hypertensive disorders of pregnancy among women with a BMI ≥ 30 Kg/m^2^. The AUC for gestational weight gain in relation to hypertensive disorders of pregnancy was 0.63 (95% CI: 0.54–0.73, *p* < 0.01), and in relation to pre-eclampsia, it was 0.65 (95% CI: 0.54–0.76, *p* < 0.01). According to the receiver operating characteristic curve analysis, the cut-off value of weight gain during pregnancy for hypertensive disorders of pregnancy development was 3.5 kg, with a sensitivity of 0.75 and specificity of 0.49. For pre-eclampsia development, the cut-off value was 3.6 kg, with a sensitivity of 0.77 and specificity of 0.49.

## 4. Discussion

In this study, we examined the relationship between gestational weight gain and the risk of hypertensive disorders of pregnancy among primiparous women with obesity. Our analysis found a significant difference in the occurrence of hypertensive disorders of pregnancy between two groups of women: those with a BMI of 25 to <30 kg/m^2^ and those with a BMI ≥ 30 kg/m^2^. Multiple logistic regression analysis indicated that in primiparous women with a pre-pregnancy BMI of 25 to <30 kg/m^2^, the risks of hypertensive disorders of pregnancy and pre-eclampsia increase with weight gain during pregnancy. Receiver operating characteristic analysis determined the cut-off values for pregnancy weight gain in this group to be 10.5 kg for hypertensive disorders of pregnancy and 10.6 kg for pre-eclampsia. On the other hand, in women with a pre-pregnancy BMI ≥ 30 kg/m^2^, a significant association was found between gestational weight gain and the onset of hypertensive disorders of pregnancy in the multivariate analysis. However, no significant association was found between gestational weight gain and gestational hypertension or pre-eclampsia. In this same group, the receiver operating characteristic curve showed cut-off values for hypertensive disorders of pregnancy and pre-eclampsia of 3.5 kg and 3.6 kg, respectively. These findings suggest that, while weight gain is related to hypertensive disorders of pregnancy in women with a BMI of 25 to <30 kg/m^2^, for women with a BMI ≥ 30 kg/m^2^, the risk of hypertensive disorders of pregnancy may be more influenced by the BMI itself rather than by gestational weight gain.

Obesity before pregnancy, defined as a BMI ≥ 25 kg/m^2^ in Japan, is widely recognized to be associated with the occurrence of hypertensive disorders of pregnancy [18]. Despite the high priority given to preventing hypertensive disorders of pregnancy in Japan [19], there is limited evidence on the appropriate weight gain during pregnancy for Japanese women with obesity. Hirooka-Nakama et al. suggested, based on the Japan Society of Obstetrics and Gynecology registry system, that weight gain between 0.0 and 11.5 kg for overweight women (BMI 25 to <30 kg/m^2^) and weight loss for women with obesity (BMI ≥ 30 kg/m^2^) could minimize the risk of inappropriate fetal growth, such as being small for gestational age or large for gestational age [20]. However, their study found no association between gestational weight gain and hypertensive disorders of pregnancy in overweight and obese women [20]. Suzuki’s single tertiary referral center study indicated that conventional recommendations for weight gain during pregnancy (up to 7 kg) for overweight Japanese women might be more stringent than those in other countries [21]. Due to the lack of evidence on the optimal gestational weight gain to reduce the risk of hypertensive disorders of pregnancy among pregnant Japanese women with obesity, the Japan Society of Obstetrics and Gynecology guidelines (revised in 2020) recommended “personalized counseling between physicians and pregnant women” for those with a BMI ≥ 25 kg/m^2^ [22]. To the best of our knowledge, our study is the first in Japan to provide guidelines for appropriate weight gain during pregnancy to prevent hypertensive disorders of pregnancy in both overweight women (BMI 25 to <30 kg/m^2^) and women with obesity (BMI ≥ 30 kg/m^2^).

In the current study, we focused exclusively on primiparous women. It is well-known that first-time deliveries increase the risk of hypertensive disorders of pregnancy, while subsequent pregnancies tend to reduce the occurrence of hypertensive disorders of pregnancy due to improved implantation or placentation [23] and enhanced maternal cardiovascular adaptation [24,25,26]. This enhanced adaptation involves increased end-diastolic blood and stroke volume, as well as decreased vascular resistance [24], leading to reduced mean arterial pressure and arterial stiffness. Considering societal changes leading to an increase in maternal age, in Japan, between 1990 and 2015, the rate of advanced maternal age rose from 7.7% to 29.0%, and the ratio of first-time pregnancy also grew from 27.0% to 30.7% [16]. Therefore, taking into account recent societal backgrounds, our results may provide valuable information for preventing hypertensive disorders of pregnancy in first-time mothers with obesity.

Various factors including abnormal placentation and environmental exposure are known to influence hypertensive disorders of pregnancy, a multifactorial disorder [4,5,27]. Our study found that not only abnormal placental invasion but also excessive weight gain could contribute to the occurrence of hypertensive disorders of pregnancy, suggesting the importance of maternal nutritional status. Recently, there has been a growing emphasis on preconception health, given its substantial influence on obstetric complications and the long-term well-being of both the mother and the offspring [28]. Notably, women with a history of hypertensive disorders of pregnancy are at an increased risk of diabetes and cardiovascular-related mortality and morbidity later in their lives [29,30]. Weight gain represents a factor that can be modified, and preconception advice on weight management can aid in modifying dietary habits during pregnancy [31,32,33,34,35,36] and postpartum in obese women. Therefore, endorsing suitable weight gain becomes an excellent personalized approach to enhance pregnancy outcomes among primiparous women with obesity.

One strength of our study is the utilization of data from two tertiary care maternal-fetal medical units, where all women who deliver are managed under a nearly uniform protocol [14]. Furthermore, our study population consisted exclusively of Japanese women, thereby eliminating the impact of ethnic variability on the observed outcomes. However, our study also had several limitations. First, as the study exclusively involved Japanese participants, it remains uncertain whether these findings can be generalized to other countries. Second, while we considered several confounding factors, we did not account for maternal nutritional status during pregnancy, which could potentially influence the development of hypertensive disorders of pregnancy or pre-eclampsia [5,32,36]. Consequently, future research should incorporate maternal nutritional status, both prior to and throughout pregnancy. For instance, methods such as the dietary inflammatory index could be used to assess the comprehensive tendency of inflammation caused by diet. Kyozuka et al. reported that a higher dietary inflammatory index, indicating a more pro-inflammatory diet, is associated with an increased risk of developing hypertensive disorders of pregnancy [32]. Third, we focused on women who are overweight and obese, thus excluding pregnant women with a BMI < 25 kg/m^2^ from the present analysis. We have previously categorized women with a BMI < 25 kg/m^2^ into four groups for analysis (<18.5, 18.5 to <20.0, 20.0 to <23.0, and 23.0 to <25.0 kg/m^2^) [12,15,35,37]. Therefore, further studies focusing on these women could lead to a better understanding of the relationship between weight gain during pregnancy and the onset of hypertensive disorders of pregnancy. Lastly, we did not address the issue of progressive edema during hypertensive disorders of pregnancy, as edema is not a diagnostic criterion for pre-eclampsia [38] and is more likely to occur in women who develop pre-eclampsia. This might cause a higher weight gain during pregnancy and consequently overestimate excessive gestational weight gain when evaluating pre-eclampsia risk. Therefore, it is challenging to determine whether increased edema in women with pre-eclampsia causes greater weight gain or whether greater weight gain contributes to pre-eclampsia.

In conclusion, there is limited information in Japan regarding the appropriate weight gain during pregnancy for women with a pre-pregnancy BMI ≥ 25.0 kg/m^2^. Our findings suggest that gestational weight gain is a potentially modifiable risk factor for hypertensive disorders of pregnancy among these women. The study results also indicate that managing weight gain during pregnancy can help prevent the development of hypertensive disorders of pregnancy in women with obesity. Based on our analysis, we recommend a cut-off value of 10.6 kg for weight gain in primiparous women with a pre-pregnancy BMI of 25.0 to < 30.3 kg/m^2^ to prevent pre-eclampsia during pregnancy. Moreover, these findings underscore the importance of appropriate preconception care for overweight and obese women, suggesting that early interventions to manage and monitor weight can be crucial in mitigating the risks associated with hypertensive disorders of pregnancy.

## Figures and Tables

**Table 1:** Basic characteristics of the participants according to BMI before pregnancy.

Variable	BMI 25 to <30 N = 331	BMI ≥ 30 N = 177	*p* Value
Maternal age, mean years (SD)	30.2 (5.3)	31.1 (5.3)	0.052 ^a^
Maternal age more than 30 years, % (*n*)	53.7	61.3	0.102 ^b^
Assisted reproductive technology, %	6.0	4.5	0.199 ^b^
Maternal smoking, %	10.3	10.8	0.089 ^b^
Diabetes mellitus, %	10.0	16.9	<0.001 ^b^
Gestational weight gain, mean (95% CI) kg	7.2 (6.4–8.0)	4.0 (3.1–4.8)	<0.001 ^a^
Obstetrics outcomes			
Hypertensive disorders of pregnancy, %	14.2	22.6	0.017 ^b^
Gestational hypertension, %	6.3	10.2	0.123 ^b^
Pre-eclampsia, %	7.9	12.4	0.093 ^b^

SD, standard deviation; BMI, body mass index; CI, confidence interval. ^a^ *p* value from student *t*-test; ^b^ *p* value from chi-squared test. Hypertensive disorders of pregnancy were further categorized into gestational hypertension and pre-eclampsia.

**Table 2:** Association between weight gain during pregnancy and risk of hypertensive disorders of pregnancy among women with pregnancy obesity, based on parity.

	BMI 25 to <30			BMI ≥ 30		
Hypertensive disorders of pregnancy						
Weight gain	OR	95% CI	*p* value	OR	95% CI	*p* value
	1.10	1.03–1.17	0.003	1.07	1.00–1.14	0.041
	aOR	95% CI	*p* value	aOR	95% CI	*p* value
	1.09	1.03–1.16	0.006	1.07	1.00–1.15	0.048
Gestational hypertension						
Weight gain	OR	95% CI	*p* value	OR	95% CI	*p* value
	1.08	0.99–1.18	0.070	1.03	0.94–1.12	0.512
	aOR	95% CI	*p* value	aOR	95% CI	*p* value
	1.07	0.98–1.17	0.124	1.03	0.95–1.13	0.464
Pre-eclampsia						
Weight gain	OR	95% CI	*p* value	OR	95% CI	*p* value
	1.10	1.01–1.19	0.023	1.09	1.00–1.18	0.046
	aOR	95% CI	*p* value	aOR	95% CI	*p* value
	1.10	1.01–1.20	0.024	1.08	0.99–1.18	0.069

BMI, body mass index; OR, odds ratio; CI, confidence interval; aOR, adjusted odds ratio. aOR was calculated by logistic regression analysis, and the model included maternal age (<30 years), maternal smoking status, gestational diabetes mellitus, and whether or not assisted reproductive technology was used for conception. Hypertensive disorders of pregnancy were further categorized into gestational hypertension and pre-eclampsia.

## Data Availability

The data that support the findings of this study are available from the corresponding author, H.K, upon reasonable request.
